# A safe and complete algorithm for metagenomic assembly

**DOI:** 10.1186/s13015-018-0122-7

**Published:** 2018-02-07

**Authors:** Nidia Obscura Acosta, Veli Mäkinen, Alexandru I. Tomescu

**Affiliations:** Helsinki Institute for Information Technology HIIT, Department of Computer Science, University of Helsinki, Helsinki, Finland

**Keywords:** Genome assembly, Contig assembly, Metagenomics, Graph algorithm, Circular walk

## Abstract

**Background:**

Reconstructing the genome of a species from short fragments is one of the oldest bioinformatics problems. Metagenomic assembly is a variant of the problem asking to reconstruct the circular genomes of all bacterial species present in a sequencing sample. This problem can be naturally formulated as finding a collection of circular walks of a directed graph *G* that together cover all nodes, or edges, of *G*.

**Approach:**

We address this problem with the “safe and complete” framework of Tomescu and Medvedev (Research in computational Molecular biology—20th annual conference, RECOMB 9649:152–163, 2016). An algorithm is called *safe* if it returns only those walks (also called *safe*) that appear as subwalk in all metagenomic assembly solutions for *G*. A safe algorithm is called *complete* if it returns all safe walks of *G*.

**Results:**

We give graph-theoretic characterizations of the safe walks of *G*, and a safe and complete algorithm finding *all* safe walks of *G*. In the node-covering case, our algorithm runs in time $O(m^2 + n^3)$, and in the edge-covering case it runs in time $O(m^2n)$; *n* and *m* denote the number of nodes and edges, respectively, of *G*. This algorithm constitutes the first theoretical tight upper bound on what can be safely assembled from metagenomic reads using this problem formulation.

## Background

One of the oldest bioinformatics problems is to reconstruct the genome of an individual from short fragments sequenced from it, called *reads* (see [1–3] for some genome assembly surveys). Its most common mathematical formulations refer to an assembly (directed) graph built from the reads, such as a *string graph* [4, 5] or a *de Bruijn graph* [6, 7]. The nodes of such a graph are labeled with reads, or with sub-strings of the reads.^1^ Standard assembly problem formulations require to find e.g., a node-covering circular walk in this graph [8], an edge-covering circular walk [8–11],^2^ a Hamiltonian cycle [12, 13] or an Eulerian cycle [7].

Real assembly graphs have however many possible solutions, due mainly to long repeated sub-strings of the genome. Thus, assembly programs used in practice, e.g., [5, 14–18], output only (partial) strings that are promised to occur in *all* solutions to the assembly problem. Following the terminology of [19], we will refer to such a partial output as a *safe* solution to an assembly problem; an algorithm outputting *all* safe solutions will be called *complete*. Even though practical assemblers incorporate various heuristics, they do have safe solutions at their core. Improving these can improve practical assembly results, and ultimately characterizing *all* safe solutions to an assembly problem formulation gives a tight upper bound on what can be reliably assembled from the reads.

We will assume here that the genome to be assembled is a node or edge-covering circular walk of the input graph, since Hamiltonian or Eulerian cycle formulations unrealistically assume that each position of the genome is sequenced exactly the same number of times. The quest for safe solutions for this assembly problem formulation has a long history. Its beginnings can be traced to [20], which assembled the paths whose internal nodes have in-degree and out-degree equal to one. The method [7] assembled those paths whose internal nodes have out-degree equal to one, with no restriction on their in-degree. Other strategies such as [9, 21, 22] are based on iteratively reducing the assembly graph, for example by contracting edges whose target has in-degree equal to one. In [19], Tomescu and Medvedev found the first safe and complete algorithms for this problem, by giving a graph-theoretic characterization of *all* walks of a graph that are common to all of its node or edge-covering circular walks. The algorithm for finding them, though proven to work in polynomial time, launches an exhaustive visit of all walks starting at each edge, and extending each walk as long as it satisfies the graph-theoretic characterization.

The present paper is motivated by *metagenomic sequencing* [23, 24], namely the application of genomic sequencing to environment samples, such as soils, oceans, or parts of the human body. For example, metagenomic sequencing helped discover connections between bacteria in the human gut and bowel diseases [25, 26] or obesity [27]. A metagenomic sample contains reads from all the circular bacterial genomes present in it.

**Fig. 1:**
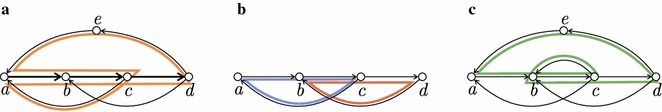
Node-safe walks. In **a**, the walk (*a*, *b*, *c*, *d*) is node-safe, because every circular walk covering node *e* contains (*a*, *b*, *c*, *d*) as sub-walk (we draw one such circular walk in orange). In **b**, the walk (*a*, *b*, *c*, *d*) is not node-safe, because the graph admits two circular walks covering all nodes (in blue and red) that do not contain it as sub-walk; it does not satisfy condition (*b*) of Theorem 2. In **c** the walk (*a*, *b*, *c*, *d*) is not safe because there is a node-covering circular walk not containing it as sub-walk (in green); it does not satisfy condition (*a*) of Theorem 2

Because of the multiple genomes present in the sample, one can no longer formulate a solution for the metagenomic assembly problem as a *single* circular walk covering all the nodes or edges. A natural analog is to find a *collection* of circular walks of an assembly graph (i.e., the circular bacterial genomes), which *together* cover all the nodes, or edges, of the graph (i.e., they together explain all the reads). In general, we do not know how many bacterial species are in the sample, so we cannot place any bound on the number of circular walks. Hence, in our above formulation they can be any arbitrary number. See the next section for formal definitions, and Fig. 1 for a simple example.

It can be easily verified that the walks from [7, 9, 20–22]—which are safe for single circular covering walks—are also safe for this metagenomic problem formulation. However, even though many practical metagenomic assemblers exist, e.g., [28–34], no other safe solutions are known for this problem formulation.

In this paper we solve this problem, by giving a graph-theoretic characterization of *all* walks *w* of a graph *G* such that for any metagenomic assembly solution *R* of *G*, *w* is a sub-walk of some circular walk in *R*. As opposed to the exhaustive search strategy from [19], in this paper we devise a new type of safe and complete algorithm for which we can tightly bound the running time. This works by outputting one solution to the metagenomic assembly problem, and then marking all its sub-walks that satisfy our characterization. The algorithm for the node-covering case can be implemented with a complexity of $O(m^2 + n^3)$, and the one for the edge-covering case with a complexity of $O(m^2n)$; *n* and *m* denote the number of nodes and edges, respectively, of the input graph. This is achieved by pre-processing the graph and the metagenomic assembly solution so that for each of its sub-walks we can check in constant time if they satisfy our characterization.

We then show how to modify this algorithm to explicitly output all *maximal* safe walks (i.e., not contained in another safe walk), with a logarithmic slowdown, namely $O(m^2 + n^3\log n)$ and $O(m^2n\log n)$, respectively. This is based on constructing a suffix-tree from the metagenomic assembly solution, and traversing it using suffix links.

### Related work

This paper also falls into a broad line of research dealing with real-life problems that cannot model sufficiently well the real data. Other strategies for dealing with these in practice are to enumerate all solutions (as done e.g. in [35]), or to find the best *k* solutions (see e.g., [35, 36]).

Other bioinformatics studies that considered partial solutions common to all solutions are [37, 38], which studied base-pairings common to all optimal alignments of two biological sequences under edit distance. In combinatorial optimization, safety has been studied under the name of *persistency*. For a given problem on undirected graphs, the *persistent* nodes or edges are those present in all solutions to the problem [39]. This question was first studied for the maximum matching problem of a bipartite graph [39], and later developed for more general assignment problems [40]. Later papers studied persistent nodes present in all maximum stable sets of a graph [41], or persistent edges present in all traveling salesman solutions on a particular class of graphs where the problem is polynomially solvable [42].

Persistency has been recently generalized from single edges to sets of edges by the notions of *transversal* and *blocker* [43]: a *d-traversal* is a set of edges intersecting any optimum solution in at least *d* elements, and a *d-blocker* is a subset of edges whose removal deteriorates the value of the optimum solution by at least *d*. These notions have been studied for maximum matchings in arbitrary graphs [43], maximum stable sets [44], or for the maximum weight clique problem [45]. The problem closest to ours is the one of finding a minimum-cardinality *d*-transversal of all *s*–*t* paths in a directed graph, shown to be polynomially solvable in [44].

## Preliminaries and main definitions

In this paper by *graph* we always mean a directed graph. The number of *nodes* and *edges* in a graph *G* are denoted by *n* and *m*, respectively. We do not allow parallel edges, but allow self-loops and edges of opposite directions. For any node $v \in V(G)$, we use $N^-(v)$ to denote its set of in-neighbors, and $N^+(v)$ to denote its set of out-neighbors.

A *walk* in a graph is a sequence $w = (v_0,e_0,v_1,e_1,\dots ,v_t,e_t,v_{t+1})$ where $v_0,\dots ,v_{t+1}$ are *nodes*, and each $e_i$ is an *edge* from $v_i$ to $v_{i+1}$ ($t \ge -1$). The *length* of *w* is its number of edges, namely $t+1$. Walks of length at least one are called *proper*. Sometimes, we may omit explicitly writing the edges of *w*, and write only its nodes, i.e., $w = (v_0,v_1,\dots ,v_t,v_{t+1})$. We will also say that an edge $(x,y) \in E(G)$ is a walk of length 1.

A *path* is a walk where all nodes are distinct. A walk whose first and last nodes coincide is called a *circular* walk. A path (walk) with first node *u* and last node *v* will be called a path (walk) *from* *u* *to* *v*, and will be denoted as *u-v path (walk)*. A *cycle* is a circular walk of length at least one (a self-loop) whose first and last nodes coincide, and all other nodes are distinct. If $u = v$, then by *v–u path* we denote a cycle passing through *v*. A walk is called *node-covering* or *edge-covering* if it passes through each node, or respectively edge, of the graph at least once.

Given a non-circular walk $w = (v_0,v_1,\dots ,v_{t-1})$ and a walk $w' = (u_0,\dots ,u_{d-1})$, we say that $w'$ is a sub-walk of *w* if there exists an index in *w* where an occurrence of $w'$ starts. If $w = (v_0,v_1,\dots ,v_{t-1},v_t = v_0)$ is a circular walk, then we allow $w'$ to “wrap around” *w*. More precisely, we say that $w'$ is a *sub-walk* of *w* if $d \le t$ and there exists an index $i \in \{0,\dots ,t-1\}$ such that $v_i = u_0$, $v_{i+1 \bmod t} = u_1$, ..., $v_{i+d-1 \bmod t} = u_{d-1}$.

The following reconstruction notion captures the notion of solution to the metagenomic assembly problem.

### **Definition 1**

(*Node-covering metagenomic reconstruction*) Given a graph *G*, a *node-covering metagenomic reconstruction of G* is a collection *R* of circular walks in *G*, such that every node of *G* is covered by some walk in *R*.

The following definition captures the walks that appear in all node-covering metagenomic reconstructions of a graph (see Fig. 1 for an example).

### **Definition 2**

(*Node-safe walk*) Let *G* be a graph with at least one node-covering metagenomic reconstruction, and let *w* be a walk in *G*. We say that *w* is a *node-safe walk in G* if for any node-covering metagenomic reconstruction *R* of *G*, there exists a circular walk $C \in R$ such that *w* is a sub-walk of *C*.

We analogously define *edge-covering metagenomic reconstructions* and *edge-safe walks* of a graph *G*, by replacing node with edge throughout. Reconstructions consisting of exactly one circular node-covering walk were considered in [19]. The following notion of node-omnitig was shown in [19] to characterize the node-safe walks of such reconstructions.

### **Definition 3**

(*Node-omnitig*, [19]) Let *G* be a graph and let $w = (v_0,e_0,v_1,e_1,\dots ,v_t,e_t,v_{t+1})$ be a walk in *G*. We say that *w* is a *node-omnitig* if both of the following conditions hold:

- for all $1 \le i \le j \le t$, there is no proper $v_j$–$v_i$ path with first edge different from $e_j$, and last edge different from $e_{i-1}$, and
- for all $0 \le j \le t$, the edge $e_j$ is the only $v_j$–$v_{j+1}$ path.

### **Theorem 1**

[19]* Let* *G be a strongly connected graph different from a cycle. A walk* *w* in *G is a sub-walk of all node-covering circular walks in* *G if and only if* *w is a node-omnitig*.

Observe that the circular walks in a node-covering metagenomic reconstruction of a graph *G* stay inside its strongly connected components (because e.g., the graph of strongly connected components is acyclic). Likewise, a graph *G* admits at least one edge-covering metagenomic reconstruction if and only if *G* is made up of a disjoint union of strongly connected graphs. Thus, in the rest of the paper we will assume that the input graphs are strongly connected.

## Characterizations of safe walks

In this section we give characterizations of node- and edge-safe walks. The difference between our characterization below and Theorem 1 lies in the additional condition (b). Note that (b) refers to cycles, whereas the elements of a node-covering metagenomic reconstruction are arbitrary circular walks; this is essential in our algorithm from the next section.

### **Theorem 2**

*Let* *G* *be a strongly connected graph. A walk* $w = (v_0,e_0,v_1,e_1,\dots ,v_t,e_t,v_{t+1})$ *in* *G* *is a node-safe walk in* *G* *if and only if the following conditions hold:*

- *w is a node-omnitig, and*
- *there exists* $x \in V(G)$* such that* *w is a sub-walk of all cycles passing through* *x*.

### *Proof*

$(\Rightarrow )$ Assume that *w* is safe. Suppose first that (a) does not hold, namely that *w* is not an omnitig. This implies that either (i) there exists a proper $v_j$-$v_i$ path *p* with $1\le i \le j \le t$ with first edge different from $e_j$, last edge different from $e_{i-1}$, or (ii) there exists *j*, $0 \le j \le t$, and a $v_j$-$v_{j+1}$ path $p'$ different from the edge $e_j$.

Suppose (i) is true. For any node-covering metagenomic reconstruction *R* of *G*, and any circular walk $C \in R$ such that *w* is a sub-walk of *C*, we replace *C* in *R* by the circular walk $C'$, not containing *w* as sub-walk, obtained as follows. Whenever *C* visits *w* until node $v_j$, $C'$ continues with the $v_j$–$v_i$ path *p*, then it follows $(v_i,e_i,\dots ,e_{j-1},v_j)$, and finally continues as *C*. Since *p* is proper, and its first edge is different from $e_j$ and its last edge is different from $e_{i-1}$, the only way that *w* can appear in $C'$ is as a sub-walk of *p*. However, this implies that both $v_j$ and $v_i$ appear twice on *p*, contradicting the fact that *p* is a $v_j$–$v_i$ path. Since each such circular walk $C'$ covers the same nodes as *C*, the collection $R'$ of circular walks obtained by performing all such replacements is also a node-covering metagenomic reconstruction *G*. This contradicts the safety of *w*.

Suppose (ii) is true. As above, for any node-covering metagenomic reconstruction *R* and any $C \in R$ containing *w* as sub-walk, we replace *C* with the circular walk $C'$ obtained as follows. Whenever *C* traverses the edge $e_j$, $C'$ traverses instead $p'$, and thus covers the same nodes as *C*, but does not contain *w* as sub-walk. This also contradicts the safety of *w*.

Suppose now that (b) does not hold, namely, that for every $x \in V(G)$, there exists a cycle $c_x$ passing through *x* such that *w* is not a sub-walk of $c_x$. The set $R = \{c_x \text{: } x \in V(G)\}$ is a node-covering metagenomic reconstruction of *G* such that *w* is not a sub-walk of any of its elements. This contradicts the safety of *w*.

$(\Leftarrow )$ Let *R* be a node-covering metagenomic reconstruction of *G*, and let $C \in R$ be a circular walk covering the vertex *x*. If *C* is a cycle, then (b) implies that *w* is a sub-walk of *C*, from which the safety of *w* follows.

Otherwise, let *G*[*C*] be the subgraph of *G* induced by the edges of *C*. Clearly, *C* is a node-covering circular walk of *G*[*C*], and thus *G*[*C*] is strongly connected. Moreover, we can argue that *w* is a node-omnitig in *G*[*C*], as follows. By taking the shortest proper circular sub-walk of *C* passing through *x* we obtain a cycle $\widetilde{C}$ passing through *x*. From (b), we get that *w* is a sub-walk of $\widetilde{C}$. Since all edges of $\widetilde{C}$ appear in *G*[*C*], then also all edges of *w* appear in *G*[*C*] and thus *w* is a walk in *G*[*C*]. The two conditions from the definition of node-omnitigs are preserved under removing edges from *G*, thus *w* is a node-omnitig also in *G*[*C*]. By applying Theorem 1 to *G*[*C*] we obtain that *w* is a sub-walk of all node-covering circular walks of *G*[*C*], and in particular, also of *C*. We have thus shown that for every node-covering metagenomic reconstruction *R* of *G*, there exists $C \in R$ such that *w* is a sub-walk of *C*. Therefore, *w* is a node-safe walk for *G*. $\square$

The following statement is a simple corollary of condition (b) from Theorem 2.

### **Corollary 3**

*Let* *G be a strongly connected graph, and let* *w be a safe walk in* *G*.* The*n *w is either a path or a cycle.*

We now give the analogous characterization of edge-safe walks. We first recall the analogous definition of edge-omnitigs from [19]. This is the same as Definition 3, except that the second condition is missing.

### **Definition 4**

(*Edge-omnitig,* [19]) Let *G* be a graph and let $w = (v_0,e_0,v_1,e_1,\dots ,v_t,e_t,v_{t+1})$ be a walk in *G*. We say that *w* is an *edge-omnitig* if for all $1 \le i \le j \le t$, there is no proper $v_j$–$v_i$ path with first edge different from $e_j$, and last edge different from $e_{i-1}$.

In [19], it was proven an equivalent of Theorem 1 in terms of edges, stating that edge-omnitigs characterize the walks of a strongly connected graph *G* that are sub-walks of all edge-covering circular walks of *G*. Our characterization of the edge-safe walks considered in this paper is:

### **Theorem 4**

*Let* *G* *be a strongly connected graph. A walk* $w = (v_0,e_0,v_1,e_1,\dots ,v_t,e_t,v_{t+1})$ *in* *G* *is an edge-safe walk in* *G* *if and only if the following conditions hold:*

- *w is an edge-omnitig, and*
- *there exists* $e \in E(G)$* such that* *w is a sub-walk of all cycles passing through* *e*.

Theorem 4 could be proved by carefully following the proof outline of Theorem 2. However, below we give a simpler proof, by reducing Theorem 4 to the node-covering case in the graph *S*(*G*) obtained from *G* by sub-dividing every edge once.

Given a graph *G*, we let *S*(*G*) denote the graph obtained from *G* by subdividing each edge once. Namely, each edge (*u*, *v*) of *G* is replaced by two edges $(u,x_{uv})$, and $(x_{uv},v)$, where $x_{uv}$ is a new node for every edge. Observe that the nodes $x_{uv}$ have exactly one in-neighbor, *u*, and exactly one out-neighbor, *v*. We can analogously define this operation for a walk *w* in *G*, and then consider the walk *S*(*w*) in *S*(*G*).

### Proof of Theorem 4

**Fig. 2:**
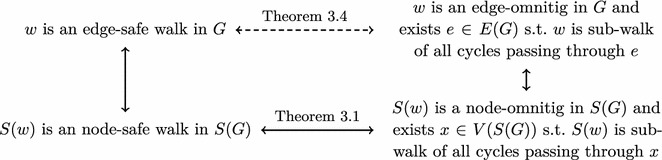
The proof outline of Theorem 4

The proof follows the outline given in Fig. 2. We first argue that *w* is an edge-safe walk in *G* if and only if *S*(*w*) is a node-safe walk in *S*(*G*). Indeed, observe that the edge-covering metagenomic reconstructions of *G* are in bijection with the node-covering metagenomic reconstructions of *S*(*G*), the bijection being $R \mapsto \{S(C) \text{: } C \in R\}$. Moreover, *w* is a sub-walk of a walk *C* in *G* if and only if *S*(*w*) is a sub-walk of *S*(*C*) in *S*(*G*). Therefore, *w* is an edge-safe walk in *G* if and only if *S*(*w*) is a node-safe walk in *S*(*G*).

It remains to show that *w* satisfies conditions (a) and (b) of Theorem 4 for *G* if and only if *S*(*w*) satisfies conditions (a) and (b) of Theorem 2 for *S*(*G*).

*Condition (a):* It immediately follows from the definition that if *S*(*w*) is a node-omnitig in *S*(*G*) then *w* is an edge-omnitig in *G*. Assume now that *w* is an edge-omnitig in *G*. By the construction of *S*(*G*) and *S*(*w*), between any two consecutive nodes of *S*(*w*) there can be only one path in *S*(*G*) (namely, the edge connecting the two nodes). Therefore, *S*(*w*) is a node-omnitig in *S*(*G*).

*Condition (b):* Suppose that there exists an edge $e = (u,v) \in E(G)$ such that all cycles in *G* passing through *e* contain *w* as sub-walk. Then by construction all cycles in *S*(*G*) passing through $x_{uv} \in V(S(G))$ also contain *S*(*w*) as sub-walk. Conversely, suppose that there exists a node $x \in V(S(G))$ such that all cycles in *S*(*G*) passing through *x* contain *S*(*w*) as sub-walk. If *x* is a node of the type $x_{uv}$ for some edge (*u*, *v*) of *G*, then it also holds that all cycles in *G* passing through $(u,v) \in E(G)$ contain *w* as sub-walk. Otherwise, if $x \in V(G)$, then let (*x*, *y*) be an arbitrary edge of *G* out-going from *x*; this exists because *G* is strongly connected. We claim that all cycles in *G* passing through $(x,y) \in E(G)$ contain *w* as sub-walk. Indeed, let $z_{xy}$ be the node of *S*(*G*) corresponding to the edge (*x*, *y*). The set of cycles of *S*(*G*) passing through $z_{xy}$ is a subset of the set of cycles of *S*(*G*) passing through *x*. Therefore, all cycles of *S*(*G*) passing through $z_{xy}$ contain *S*(*w*) as sub-walk. We have now reduced this case to the previous one, when *x* is a node of the type $x_{uv}$ for some edge (*u*, *v*) of *G*, and the claim follows. $\square$

## The algorithm for finding all node-safe walks

In this section we give an algorithm for finding all node-safe walks of a strongly connected graph. In the next section we show how to implement this algorithm to run in $O(m^2+n^3)$ time. Our results for edge-safe walks are analogous, and will be given in the last section.

We begin with an easy lemma stating a simple condition when a maximum overlap of two node-omnitigs is a node-omnitig.

### **Lemma 5**

*Let* *G be a graph, and let* $w = (v_0,e_0,v_1,\dots ,v_t,e_t,v_{t+1})$* be a walk of length at least two in* *G*.* We have that* *w is a node-omnitig if and only if* $w_1 = (v_0,e_0,v_1,\dots ,v_t)$* and* $w_2 = (v_1,e_1,v_2,\dots ,v_t,e_t,v_{t+1})$* are node-omnitigs and there is no* $v_t$–$v_1$* path with first edge different than* $e_t$* and last edge different than* $e_0$.

### *Proof*

The forward implication is trivial, as by definition sub-walks of node-omnitigs are node-omnitigs. For the backward implication, since both $w_1$ and $w_2$ are node-omnitigs, then for all $0 \le j \le t$, the edge $e_j$ is the only $v_j$–$v_{j+1}$ path. Since $w_1$ is a node-omnitig, then for all $1 \le i \le j \le t-1$, there is no proper $v_j$-$v_i$ path with first edge different from $e_j$, and last edge different from $e_{i-1}$. If there is no $v_t$-$v_1$ path with first edge different than $e_t$ and last edge different than $e_0$, we obtain that *w* is a node-omnitig. $\square$

The following definition captures condition (b) from Theorem 2. Note that the walk *w* can also be a single node.

### **Definition 5**

(*Certificate*) Let *G* be a graph and let *w* be a walk in *G*. A node $x \in V(G)$ such that *w* is a sub-walk of all cycles passing through *x* is called a *certificate* of *w*. The set of all certificates of *w* will be denoted $\mathsf {Cert}(w)$.

By Theorem 2, node-safe walks are those node-omnitigs with at least one certificate. In the following lemma we relate the certificates of a node-omnitig with the certificates of its nodes. Later, in Lemma 8, we will show that the certificates of single nodes can be computed efficiently.

### **Lemma 6**

*Let* *G be a graph and let* $w = (v_0,e_0,v_1,\dots ,v_t,e_t,v_{t+1})$* be a proper node-omnitig in* *G*.* Then* $\mathsf {Cert}(w) = \mathsf {Cert}(v_0) \cap \mathsf {Cert}(v_1) \cap \cdots \cap \mathsf {Cert}(v_{t+1})$.

### *Proof*

We prove the claim by double-inclusion. The inclusion $\mathsf {Cert}(w) \subseteq \mathsf {Cert}(v_0) \cap \mathsf {Cert}(v_1) \cap \cdots \cap \mathsf {Cert}(v_{t+1})$ is trivial, since all cycles passing through a node $x \in \mathsf {Cert}(w)$ also contain each of $v_0,\dots ,v_{t+1}$.

We now prove the reverse inclusion by induction on the length of *w*. We first check the base case when *w* has length one. Assume for a contradiction that there is a cycle *C* passing through $x \in \mathsf {Cert}(v_0) \cap \mathsf {Cert}(v_1)$ and not having $w = (v_0,e_0,v_1)$ as sub-path. Then, after visiting *x*, (i) *C* first traverses $v_0$ and then reaches $v_1$ with a path different than $e_0$, or (ii) *C* first traverses $v_1$ and then $v_0$. The case (i) is immediately excluded, since *w* is a node-omnitig and $e_0$ is the only $v_0$–$v_1$. If (ii) holds, then there is a *x*-$v_1$ path $P_1$ and a $v_0$-*x* path $P_0$. However, the concatenation of $P_0$ with $P_1$ is a $v_0$-$v_1$ path different than the edge $e_0$, which again contradicts the fact that *w* is a node-omnitig.

We now use the inductive hypothesis to show that if $x \in \mathsf {Cert}(v_0) \cap \mathsf {Cert}(v_1) \cap \cdots \cap \mathsf {Cert}(v_{t+1})$, then $x \in \mathsf {Cert}(w)$. We partition *w* into the two walks $w_0 = (v_0,e_0,v_1,\dots ,v_{t})$ and $w_t = (v_t,e_t,v_{t+1})$. By induction, since $x \in \mathsf {Cert}(v_0) \cap \mathsf {Cert}(v_1) \cap \cdots \cap \mathsf {Cert}(v_t)$ we have $x \in \mathsf {Cert}(w_0)$. Analogously, since $x \in \mathsf {Cert}(v_t) \cap \mathsf {Cert}(v_{t+1})$, we have $x \in \mathsf {Cert}(w_t)$. Since $v_t$ is a node in both $w_0$ and $w_t$, then any cycle passing through *x*, once it passes through $w_0$ it must continue passing through $w_t$. Therefore, any cycle passing through *x* passes also through *w*, and hence $x \in \mathsf {Cert}(w)$. $\square$

Given a circular walk $C = (v_0,e_0,v_1,\dots ,v_{d-1},e_{d-1},v_d = v_0)$, $i \in \{0,\dots ,d-1\}$ and $k \in \{0,\dots ,d\}$, we denote by *C*(*i*, *k*) the sub-walk of *C* starting at $v_i$ and of length *k*, that is, $C(i,k) = (v_i,e_i,v_{i+1 \bmod d},\dots ,v_{(i+k) \bmod d})$.

Algorithm 1 finds all node-safe walks of a strongly connected graph *G* (possibly with duplicates), but does not return each node-safe walk explicitly. Instead, it returns a node-covering circular walk *C* of *G* and the set of pairs (*i*, *k*) such that *C*(*i*, *k*) is a node-safe walk.

The algorithm works by scanning *C* and checking whether each sub-walk of *C* starting at index *i* and of length *k* is a node-omnitig and has at least one certificate. If so, then it stores the index *i* in a set $S_k$, for every *k*. The algorithm first deals with the case $k=1$: it first checks whether *C*(*i*, 1) is a node-omnitig (Line 7) and whether it has at least one certificate (Line 8). The case $k > 1$ is analogous. It first checks whether $C(i,k-1)$ and $C(i+1 \bmod d,k-1)$ are omnitigs (by checking the memberships $i \in S_{k-1}$ and $i+1 \bmod d \in S_{k-1}$) and that there is no path as in the definition of node-omnitig (Line 11). Then it checks whether *C*(*i*, *k*) has at least one certificate (Line 12).

In the next section we show how to pre-process *G* and *C* to perform these verifications in constant time. This algorithm can be modified to output node-safe walks also without duplicates. For clarity, we explain this idea in the proof of Theorem 13, where we also show how to output only *maximal* node-safe walks, i.e., those that are not sub-walks of any other node-safe walk.

### **Theorem 7**

*Given a strongly connected graph* *G*,* Algorithm 1 correctly computes all the node-safe walks of* *G, possibly with duplicates.*

### *Proof*

We will first prove by induction on *k* that the set $S_k$ contains all those indices *i* for which *C*(*i*, *k*) is a node-safe walk of length *k*. In the base case $k = 1$, we explicitly check if each *C*(*i*, 1) is a node-omnitig (Line 7). We also check if *C*(*i*, 1) has at least  one certificate, by checking (due to Lemma 6) whether $\mathsf {Cert}(v_i) \cap \mathsf {Cert}(v_{i+1 \bmod 1}) \ne \emptyset$ (Line 8). Thus, for each *i* we checked whether *C*(*i*, 1) is a node-safe walk (due to Theorem 2), and the claim follows for $S_1$. We assume now that the claim is true for $S_{k-1}$. For each *i*, by Lemma 5, *C*(*i*, *k*) is a node-omnitig if and only if $C(i,k-1)$ and $C(i+1 \bmod d,k-1)$ are node-omnitigs, and there is no $v_{i+k-1 \bmod d}$-$v_{i+1 \bmod d}$ path with first edge different than $e_{i+k-1 \bmod d}$ and last edge different than $e_i$. This is verified in Line 11. In Line 12 we check whether $\mathsf {Cert}(C(i,k)) \ne \emptyset$ by checking whether $\mathsf {Cert}(v_i) \cap \dots \cap \mathsf {Cert}(v_{i+k \bmod d}) \ne \emptyset$ (due to Lemma 6). Thus the claim is true for all $S_k$.

By Corollary 3, all node-safe walks of *G* are paths or cycles, thus of length at most *n*. By the definition of node-safe, they are also sub-walks of *C*. Thus for each node-safe walk *w* of *G* of length $k \le n$, there exists $i \in \{0,\dots ,d-1\}$ such that $w = C(i,k)$ and $i \in S_k$. $\square$

## An $O(m^2 + n^3)$ implementation for node-safe walks

In this section we describe the implementation of Algorithm 1. We first show how to compute the certificates of all nodes.

### **Lemma 8**

Let *G be a strongly connected graph with* *n nodes and* *m edges. We can compute the sets* $\mathsf {Cert}(x)$* for all, in time* $x \in V(G)$*O*(*mn*).

### *Proof*

We start by initializing $\mathsf {Cert}(x) = \{x\}$ for every node *x* (recall that *G* is strongly connected). We then construct the graph $G'$ by subdividing every node of *G* once. That is, we replace every node *x* of *G* with two nodes $x_{in}$ and $x_{out}$, and add the edge $(x_{in},x_{out})$ to $G'$. Moreover, for every edge (*y*, *z*) of *G*, we add to $G'$ the edge $(y_{out},z_{in})$. Observe that also $G'$ is strongly connected.

For every $x \in V(G)$, we compute $\mathsf {Cert}(x)$ as follows. We consider the graph $G'_x$ obtained from $G'$ by removing the edge $(x_\text{{in}},x_\text{{out}})$. We compute the strongly connected components of $G'_x$, in time *O*(*m*). We then iterate through all $y \in V(G) \setminus \{x\}$ and check in constant time whether $y_\text{{in}}$ and $y_\text{{out}}$ still belong to the same strongly connected component of $G'_x$. If not, then *x* belongs to all cycles of *G* passing through *y*, and thus we add *y* to $\mathsf {Cert}(x)$. This takes in total *O*(*mn*) time. $\square$

The following lemma shows how to check in constant time the first condition in the definition of node-omnitig.

### **Lemma 9**

*Let* *G be a graph with* *m edges. We can pre-process* *G* in time $O(m^2)$* and space* $O(m^2)$* such that for every two distinct edges,* $(x_1,y_1),(x_2,y_2) \in E(G)$* we can answer in* *O(1) time if there is a* $x_1$–$y_2$* path in* *G with first edge different than* $(x_1,y_1)$* and last edge different than* $(x_2,y_2)$.

### *Proof*

**Fig. 3:**
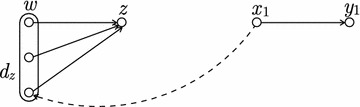
An illustration of the proof of Lemma 9

We show how to pre-compute a table $a(\cdot ,\cdot )$ of size $O(m^2)$ that for any two distinct edges $(x_1,y_1),(x_2,y_2) \in E(G)$ stores the answer to the query. See Fig. 3 for an illustration.

We iterate through all edges $(x_1,y_1) \in E(G)$, and consider the graph $G_{(x_1,y_1)}$ obtained from *G* by removing $(x_1,y_1)$. We launch a graph visit in $G_{(x_1,y_1)}$ from $x_1$ to compute to which nodes there is a path from $x_1$. By construction, any such path starts with an edge different than $(x_1,y_1)$. We then consider each node $z \in V(G)$. We first iterate once through the in-neighbors of *z* to compute how many of its in-neighbors are reachable from $x_1$ in $G_{(x_1,y_1)}$; say this number is $d_z$. We then iterate a second time through the in-neighbors of *z*, and for each in-neighbor *w*, we let $r_w$ be equal to 1 if *w* is reachable from $x_1$ in $G_{(x_1,y_1)}$, and 0 otherwise. We have that there is a $x_1$-*z* path in *G* with first edge different than $(x_1,y_1)$ and last edge different than (*w*, *z*) if and only if $d_z - r_w > 0$. Thus we set

$$\begin{aligned} a((x_1,y_1),(w,z)) =\left\{ \begin{array}{ll} true, &{} \text {if }d_z - r_w > 0, \\ false, &{} \text {if }d_z - r_w = 0. \end{array}\right. \end{aligned}$$

The complexity of this algorithm is $O(m^2)$, because for every edge $(x_1,y_1)$, we compute the set of nodes reachable from $x_1$ in time *O*(*m*), and then we process each edge of $G_{(x_1,y_1)}$ exactly two times. $\square$

Using e.g., the result of [46], we can also verify the second condition in the definition of node-omnitig in constant time.

### **Lemma 10**

*Let* *G be a graph with* *m edges, we can pre-process* *G in time* *O*(*m*)* such that for every edge* $(x,y) \in E(G)$* we can answer in* *O(1) time whether* (*x*, *y*)* is the only* *x*–*y path* .

### *Proof*

A *strong bridge* is an edge whose removal increases the number of strongly connected components of a graph (see e.g., [46]). It is easy to see that an edge $(x,y) \in E(G)$ is the only *x*–*y* path if and only if (*x*, *y*) is a strong bridge. In [46] it was shown that all strong bridges can be computed in linear time in the size of the graph, from which our claim follows. $\square$

The following lemma shows how to check in constant time condition (b) from Theorem 2. The idea is to pre-compute, for every index *i* in *C*, the smallest (i.e., left-most) index $i - n \le \ell (i) \le i$ such that $\mathsf {Cert}(v_{\ell (i)}) \cap \dots \cap \mathsf {Cert}(v_{i}) \ne \emptyset$. *C*(*i*, *k*) has a non-empty set of certificates if and only if $\ell (i)$ is at distance at least *k* to *i*, that is, $k \le i -\ell (i)$.

### **Lemma 11**

*Let* *G be a graph with* *n nodes and* *m edges, and let* $C = (v_0,e_0,v_1,\dots ,v_{d-1},e_{d-1},v_d = v_0)$* be a circular walk in* *G, with* $n \le d \le n^2$.* We can pre-process* *G* and *C in time , such that for every* $O(n^3)$ $i \in \{0,\dots ,d-1\}$* and, we can answer in* $k \in \{0,\dots ,n\}$ *O(1) time if* $\mathsf {Cert}(v_i) \cap \dots \cap \mathsf {Cert}(v_{i+k \bmod d}) \ne \emptyset$.

### *Proof*

To simplify the notation, given an integer *i*, by $v_i$ we always mean $v_{i \bmod d}$. By Lemma 8, we can compute $\mathsf {Cert}(x)$, for every $x \in V(G)$, in $O(mn) \in O(n^3)$ time. In addition to computing the index $\ell (i)$, we also compute the intersection $L_i = \mathsf {Cert}(v_{\ell (i)}) \cap \dots \cap \mathsf {Cert}(v_{i})$. Each such intersection set is stored as an array of length *n* telling in how many of $\mathsf {Cert}(v_{\ell (i)}), \dots ,\mathsf {Cert}(v_{i})$ each $x \in V(G)$ is contained; $L_i$ is non-empty if and only if there is an entry in this array with a value equaling the number of sets $\mathsf {Cert}(v_{\ell (i)}), \dots , \mathsf {Cert}(v_{i})$.

We begin by computing $\ell (i)$ and $L_i$ for $i = 0$ in a straightforward manner, by trying $\ell (i) = t = i - 1, i-2,\ldots$ as long as the resulting intersection is non-empty. Namely, we initialize $L_i = \mathsf {Cert}(v_i)$, and update it as $L_i := L_i \cap \mathsf {Cert}(v_{t})$. We keep decreasing *t* as long as $L_i$ is non-empty. If *t* reaches 0, then all sets $\mathsf {Cert}(x)$ have a common element, and the answer is “yes” for any query. Computing each intersection takes time *O*(*n*), and there are *O*(*d*) intersections to compute, giving a total of $O(dn) \in O(n^3)$ time.

For $i > 0$, we compute $\ell (i)$ as follows. We first compute $L_{i-1} \cap \mathsf {Cert}(v_i)$. If this is non-empty, then $L_i := L_{i-1} \cap \mathsf {Cert}(v_i)$ and $\ell (i) := \ell (i-1)$. By the way we store intersection sets, this can be done in *O*(*n*) time.

Otherwise, we keep increasing $\ell (i)$ by one from $t = \ell (i-1)$ until the corresponding intersection $\mathsf {Cert}(v_{t}) \cap \dots \cap \mathsf {Cert}(v_{i})$ is non-empty. We then set $L_i$ to this intersection and $\ell (i) = t$. By the way we store the intersections, it follows that we can compute the new intersection in time *O*(*n*), by scanning the current intersection and removing the elements of $\mathsf {Cert}(v_{t})$ from it, by decreasing by one the counters of its elements. Overall, such new intersections are computed at most *d* times, because for each *i* we start this scan from index $\ell (i-1)$ onwards, and always $\ell (i-1) \le \ell (i) \le i$ holds. This gives a total complexity of $O(nd) \in O(n^3)$. $\square$

We are now ready to combine these lemmas into the main theorem of this section.

### **Theorem 12**

*Algorithm 1 can be implemented to run in time* $O(m^2 + n^3)$* for any strongly connected graph with* *n nodes and* *m edges*.

### *Proof*

Any strongly connected graph admits a node-covering circular walk $C = (v_0,e_0,v_1,\dots ,v_{d-1},e_{d-1},v_d = v_0)$ of length $d \in \{n,\dots ,n^2\}$, that can be constructed in time $O(nm) \in O(n^3)$. For example, one can label the nodes of *G* as $v_1,\dots ,v_n$, start at $v_1$, then follow an arbitrary path until $v_2$ (which exists since *G* is strongly connected), and then continue from $v_2$ in the same manner. This is the same argument given in [19].

By Lemma 8, we can compute in time $O(mn) \in O(n^3)$ the sets $\mathsf {Cert}(x)$ for all $x \in V(G)$. We pre-process *G* and *C* as indicated in Lemmas 9, 10, and 11, in time $O(m^2 + n^3)$. For every length $k \in \{1,\dots ,n\}$, and every index $i \in \{0,\dots ,d-1\}$, this allows us to perform all checks in constant time. Checking membership to $S_{k-1}$ can also be done in constant time, by storing each set $S_{k}$ as a bitvector of length *d*. $\square$

**Fig. 4:**
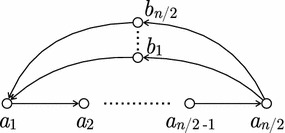
An extremal graph *G* showing that the upper bound on the complexity of Algorithm 1 from Theorem 12 is attained. The vertex set of *G* is $\{a_1,\dots ,a_{n/2},b_1,\dots ,b_{n/2}\}$. Any node- or edge-covering metagenomic reconstruction of *G* consists of circular walk(s) whose total length is $\Omega (n^2)$

In the next section we discuss how to optimize Algorithm 1 to start with a node-covering metagenomic reconstruction of minimum total length. However, there are graphs in which any node-covering metagenomic reconstruction has length $\Omega (n^2)$, see Fig. 4.

## Additional results

### Maximal node-safe walks without duplicates

In a practical genome assembly setting we want to reconstruct fragments of the genomes as long as possible. As such, we are interested only in *maximal* node-safe walks, that is, in node-safe walks that are not sub-walks of any other node-safe walk. A trivial way to obtain these is to take the output of Algorithm 1, convert it into the set of all node-safe walks of *G*, and run a suffix-tree-based algorithm for removing the non-maximal ones, in time linear in their total length. However, given a node-covering circular walk *C* of length $d \le n^2$, the total length of the node-safe walks is at most $\sum _{k = 0}^{n}kd \in O(n^4)$.

In the next theorem we show how to reduce this time complexity to $O(m^2+n^3\log n)$. The main observation is that a node-safe walk of length *k* is maximal if it is not extended into a node-safe walk of length $k+1$. We avoiding outputting duplicate maximal walks by traversing a suffix-tree built from *C* to check for previous occurrences of each walk of length *k*.

#### **Theorem 13**

*Given a strongly connected graph* *G with* *n nodes and* *m edges, Algorithm 1 can be modified to output the maximal node-safe walks of* *G explicitly and without duplicates, with a running time of* $O(m^2 + n^3\log n)$.

#### *Proof*

Let $C = (v_0,\dots ,v_{d} = v_0)$ be a node-covering circular walk *C* of *G*, of length $n \le d \le n^2$. At any position in *C* there can start the occurrence of at most one maximal node-safe walk. By Corollary 3, the length of each node-safe walk is at most *n*, thus the sum of the lengths of all maximal node-safe walks of *G* is $O(n^3)$. This implies that if we find the occurrences in *C* of all maximal node-safe walks without duplicates, then we can output all of them explicitly within the stated time bound.

A node-safe walk *w* of length *k* is maximal if no occurrence *C*(*i*, *k*) of *w* in *C* was extended left or right at step $k+1$. We can keep track of all previous occurrences of *w* in *C*, as follows. Initially, we build the suffix tree *T* of the (linear) string $C' = v_0v_1\ldots v_{d}v_1\ldots v_{n-2}\#$ over the alphabet $\Sigma = V(G) \cup \{\#\}$, where $\#$ is a new symbol. This takes time linear in the size of $C'$ and in the alphabet size $|\Sigma | = n$, thus $O(n^2)$ [47]. As we scan *C* for a length $k+1 \in \{1,\dots ,n\}$, we maintain, as we discuss below, a pointer in *T* to the node $u_i$ such that the label of the path from the root to $u_i$ spells *C*(*i*, *k*). In $u_i$ we store the information of whether any occurrence the walk $w = C(i,k)$ was extended at step $k+1$.

**Fig. 5:**
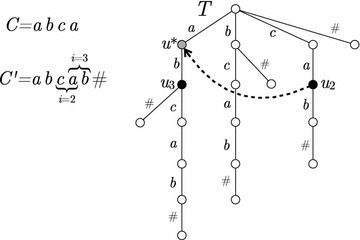
Illustration of the proof of Theorem 13; we are scanning *C* with $k = 2$. We illustrate the algorithm using the *suffix trie* of $C'$: the suffix tree is obtained by compacting the unary paths into single edges, and then many of the suffix links become implicit; we draw the suffix-link from $u_2$ to $u^*$ with a dashed arrow. Following an implicit suffix link needs to be simulated using explicit suffix link from a parent. The cost of this can be amortized to the descending in the tree

As we advance from *i* to $i+1$, we follow a so-called *suffix-link* in *T* to move to the node $u^*$ such that the label from the root of *T* to $u^*$ spells $C(i+1,k-1)$ (i.e., *C*(*i*, *k*) with its first character removed). For a detailed discussion on the properties of the suffix tree, see e.g., [48]. We then follow the normal tree edge exiting from $u^*$ labeled $v_{i+1 \bmod d}$. We thus advance to the node $u_{i+1}$ of *T* such that the path from the root to $u_{i+1}$ spells $C(i+1,k)$. See Fig. 5 for an illustration. After traversing once *C* at step $k+1$ and detecting which node-safe walks of length *k* are maximal, we traverse *C* again to output these node-safe walk.

After building the suffix tree using [47], the children of each node are organized in lexicographic order. Descending in the tree takes at most $O(\log (|\Sigma |)) = O(\log n)$ time per step for binary searching the first character of each edge. Following suffix links can be be amortized to the descending operations [48]. Thus, the above additional phase takes time $O(n^3\log n)$. The pre-computations needed in the proof of Theorem 12 take time $O(m^2 + n^3)$, from which the claimed time complexity bound follows. $\square$

### The algorithm for finding edge-safe walks

In this section we adapt Algorithm 1 and its implementation to find edge-safe walks, as characterized by Theorem 4. This will result in an algorithm running in time $O(m^2n)$. The proof of the following theorem is entirely analogous to the node-safe case.

#### **Theorem 14**

*Let* *G be a strongly connected graph with* *n nodes and* *m edges. In time we can output an edge-covering circular walk* $O(m^2n)$ *C, and the set of all pairs* (*i*, *k*)* such that* *C*(*i*, *k*)* is an edge-safe walk of* *G*.

#### *Proof*

The proof is analogous to the node-safe case, and thus we briefly sketch the differences. In the edge-covering case, the set of certificates of a walk *w* consists of the edges *e* such that all cycles passing through *e* contain *w* as sub-walk. Analogously to Lemma 6, we have that the set of certificates of a walk *w* equals the intersection of the sets of certificates of its individual edges. The algorithm for the edge-safe case is that same as Algorithm 1, with the difference that we now start with an edge-covering circular walk *C* and we do not check anymore that each *C*(*i*, 1) is the only $v_i$–$v_{i+1}$ path.

By the same argument given in the proof of Theorem 12, such a circular walk *C* has length at most *mn* and can be found in time *O*(*mn*). The certificates of all edges can be similarly computed in time $O(m^2)$ (now there is no need to subdivide nodes into single edges). Lemma 9 can be applied verbatim without modifications. The analog of Lemma 11 now starts with an edge-covering circular walk *C* of length *O*(*mn*). The only difference in its proof is that the sets of certificates now have size at most *m*, thus their intersection takes time *O*(*m*). This implies that we can pre-compute *G* and *C* in time $O(m^2n)$.

After this pre-processing phase, the algorithm itself works in time $O(mn^2)$, since the edge-covering circular walk *C* has length *O*(*mn*). $\square$

With a proof identical to the one of Theorem 13, we also obtain the following result.

#### **Theorem 15**

*Given a strongly connected graph* *G with* *n nodes and* *m edges, we can output the maximal edge-safe walks of* *G explicitly and without duplicates, in time of* $O(m^2n\log n)$.

### Optimizations to the algorithms

A trivial way to optimize Algorithm 1 is to start with a node-covering circular walk of minimum length. However, this is an NP-hard problem, since *G* has a node-covering circular walk of length *n* if and only if *G* is Hamiltonian. Observe though that instead of a single node-covering circular walk, we can start with a node-covering metagenomic reconstruction possibly consisting of multiple circular walks, and apply Algorithm 1 to each walk in the reconstruction. This is correct by definition, since node-safe walks are sub-walks of some walk in any node-covering metagenomic reconstruction.

Finding a node-covering metagenomic reconstruction whose circular walks have minimum total length can be solved with a minimum-cost circulation problem (see e.g., [49, 50] for basic results on minimum-cost circulations). From *G*, we construct the graph $G'$ by subdividing every node of *G* once (recall the construction from Lemma 8). We set demand 1 and cost 0 on each edge $(x_\text{{in}},x_\text{{out}})$, with $x \in V(G)$. On all edges corresponding to original edges of *G* we set demand 0 and cost 1. A circulation *f* in $G'$ satisfying the demands can be decomposed into cycles, which form a node-covering metagenomic reconstruction in *G*. The total length of these cycles in *G* equals the cost of *f*. Since $G'$ has no capacities, a minimum-cost circulation in $G'$ can be found in time $O(n\log U(m + n\log n))$, where *U* is the maximum value of a demand, using the algorithm of Gabow and Tarjan [51]. All demands in $G'$ are 1, thus this bound becomes $O(nm + n^2\log n)$.

In the algorithm for finding all edge-covering circular walks, we need to find an edge-reconstruction whose circular walks have minimum total length. This can be solved as above, without subdividing the nodes of *G*. We add to every edge the demand 1 and cost 1 and then compute a minimum-cost circulation. The decomposition of the optimal circulation into cycles forms an edge-reconstruction of *G*.

## Conclusions and future work

We consider [19] and the present work as starting points for characterizing all safe solutions for natural assembly problem formulations, and thus obtaining safe and complete algorithms.

As future work, we plan to study formulations where the assembly solution is made up of non-circular covering walks, or where the assembly solutions consist of a given number of covering walks (e.g., a given number of chromosomes). In terms of real graph instances, the worst-case complexity of our algorithm may be prohibitive, and thus improving it is an important problem.

We also leave for future work an idealized experimental study similar to what was done for the single genome case in [19]. This compared the lengths and biological content of some classes of safe solutions known in the literature, on de Bruijn graphs constructed from error-free, single-stranded simulated reads.

The ultimate goal of a “safe and complete” approach is to be adapted to the peculiarities of real data, such as sequencing errors, insufficient sequencing coverage, reverse complements. However, our belief is that we first need a clean and solid theoretical foundation, which can later be extended, or weakened, to account for such features.

## Footnotes

1 We refer the reader to [4–7] for definitions of string graphs and de Bruijn graphs, as they are not essential to this paper.

2 Node- and edge-covering walks usually refer to node- and edge-centric de Bruijn graphs, respectively. In the node-centric de Buijn graph, all *k*-mers in the reads are nodes of the graph, and edges are added between all *k*-mers that have a suffix-prefix overlap of length $k-1$. In the edge-centric de Bruijn graph, it is further required that the $k+1$-mer obtained by overlapping the two *k*-mers of an edge also appears in the reads. Thus for edge-centric de Bruijn graphs it reasonable to require that the walk covers all edges, because all edges also appear in the reads; this may not be the case for node-centric de Bruijn graphs.
